# The effect of an integrated palliative care intervention on quality of life and acute healthcare use in patients with COPD: Results of the COMPASSION cluster randomized controlled trial

**DOI:** 10.1177/02692163231165106

**Published:** 2023-03-31

**Authors:** Johanna Broese, Rianne MJJ van der Kleij, Els ML Verschuur, Huib AM Kerstjens, Ewald M Bronkhorst, Yvonne Engels, Niels H Chavannes

**Affiliations:** 1 Department of Public Health and Primary Care, Leiden University Medical Centre, Leiden, The Netherlands; 2 Lung Alliance Netherlands, Amersfoort, The Netherlands; 3 Department of Respiratory Medicine and Tuberculosis, University of Groningen and University Medical Centre Groningen, Groningen, The Netherlands; 4 Health Evidence, Radboud University Medical Centre, Nijmegen, The Netherlands; 5 Department of Anaesthesiology, Pain and Palliative Medicine, Radboud University Medical Centre, Nijmegen, The Netherlands

**Keywords:** COPD, palliative care, clinical effectiveness, quality of life, cluster randomized controlled trial

## Abstract

**Background:**

COPD causes high morbidity and mortality, emphasizing the need for palliative care.

**Aim:**

To assess the effectiveness of palliative care in patients with COPD.

**Design:**

Cluster randomized controlled trial (COMPASSION study; Netherlands Trial Register (NTR): NL7644, 07-04-2019). Healthcare providers within the intervention group were trained to implement palliative care components into routine COPD care. Patients completed questionnaires at baseline, after 3 and 6 months; medical records were assessed after 12 months. The primary outcome was quality of life (FACIT-Pal). Secondary outcomes were anxiety, depression, spiritual well-being, satisfaction with care, acute healthcare use, documentation of life-sustaining treatment preferences and place of death. Generalized linear mixed modelling was used for analyses.

**Setting:**

Eight hospital regions in the Netherlands.

**Participants:**

Patients hospitalized for an acute exacerbation of COPD and positive ProPal-COPD score.

**Results:**

Of 222 patients included, 106 responded to the questionnaire at 6 months. Thirty-six of 98 intervention patients (36.7%) received the intervention. Intention-to-treat-analysis showed no effect on the primary outcome (adjusted difference: 1.09; 95% confidence interval: −5.44 to 7.60). In the intervention group, fewer intensive care admissions for COPD took place (adjusted odds ratio: 0.21; 95% confidence interval: 0.03–0.81) and strong indications were found for fewer hospitalizations (adjusted incidence rate ratio: 0.69; 95% confidence interval: 0.46–1.03).

**Conclusions:**

We found no evidence that palliative care improves quality of life in patients with COPD. However, it can potentially reduce acute healthcare use. The consequences of the COVID-19 pandemic led to suboptimal implementation and insufficient power, and may have affected some of our findings.

**What is already known about the topic?**

- Patients in advanced stages of COPD suffer from high symptom burden, limited physical functioning and low quality of life.
- In oncological patients, timely initiation of palliative care alongside usual care improves quality of life and reduces healthcare use.

**What this paper adds**

- We did not find improvements in quality of life, but saw fewer intensive care admissions and a trend towards fewer hospital admissions in intervention group patients with advanced COPD.
- Study power was insufficient and not all patients received the intended palliative care intervention elements, possibly hampering reliable measurement of the clinical effectiveness.

**Implications for practice, theory or policy**

- Quality of life is a broad construct and may be difficult to target in patients with advanced organ failure; Future studies should consider a more proximal outcome measure, for example, coping with COPD.
- Lower acute healthcare use reduces healthcare costs and this is a relevant secondary outcome parameter to society as a whole; This finding needs further exploration.

## Introduction

Chronic obstructive pulmonary disease (COPD) causes considerable morbidity and is the third leading cause of death worldwide.^1^ As the disease progresses, acute exacerbations occur more frequently, requiring hospital admissions.^2^ Many patients in advanced stages suffer from severe breathlessness and other problems such as fatigue, anxiety, depression, social isolation and existential suffering.^3,4^ Their symptom burden and functional status are similar to those of patients with lung cancer and severely affect their quality of life.^5^

In patients with cancer, quality of life can be improved and healthcare use reduced by timely initiation of palliative care.^6^ Palliative care aims to enhance quality of life by addressing physical, psychological, social and spiritual problems.^7^ In addition, it endeavours to tailor patient care to their needs and preferences through advance care planning and care coordination. Patients with advanced COPD may equally benefit from palliative care.^6,8^ However, the evidence of the effectiveness of palliative care for this patient group is still scarce.

In a recent systematic review, only 4 out of 20 palliative care interventions in COPD had been evaluated in a powered controlled trial, and the effects on health outcomes remained inconclusive.^9^ Furthermore, guidelines recommend palliative care delivery by ‘generalists’ (i.e. respiratory care providers) in the first place, and only specialist palliative care involvement in case of complexity,^10,11^ but the integration of palliative care elements into routine COPD care (integrated palliative care),^12^ has hardly been studied.

Therefore, in the COMPASSION study, in half of the participating hospital regions, primary and secondary healthcare providers were trained to integrate palliative care components into routine COPD care. We assessed the effect on quality of life, emotional and spiritual well-being, acute healthcare use and place of death of patients with COPD. We hypothesized that intervention group patients would score better on quality of life and well-being, use less acute healthcare, and have a lower rate of in-hospital deaths than patients of hospitals in the control group.

## Methods

### Design

A cluster randomized controlled trial was performed. A detailed study protocol has been published previously.^13^

### Setting

This study took place in pulmonary care departments of eight hospitals in the Netherlands, that collaborated with affiliated general practitioners, primary care nurses and palliative care consultation teams, further referred to as ‘hospital regions’ or ‘clusters’.

### Randomization

Hospital regions were randomized to the intervention or control condition (four clusters in each group) by an independent statistician, stratified by the number of COPD-related hospital admissions per year.

### Intervention

An integrated palliative care intervention was developed following national guidelines, literature and stakeholders’ input and comprised (1) palliative care conversations tailored to the patient’s needs, (2) care coordination and continuity and (3) aftercare if a patient had died (Table 1). To optimize uptake of the intervention in practice, an implementation strategy was developed (Table 1). Primary and secondary healthcare providers from the intervention group were provided with an online toolbox, received two training sessions, and received implementation guidance. Healthcare providers in the control group provided care as usual and were offered training after the formal study had ended.

**Table 1.** Description of the implementation strategy and integrated palliative care intervention of the Compassion study. Adapted from Broese et al.^13^

Components	Content of the component
*Implementation strategy*	
Formation of regional intervention group	Multidisciplinary regional team consisting of pulmonologists, general practitioners, COPD nurses and palliative care nurses
Access to online toolbox	Website with information and guidance on the core elements of palliative care in COPD, including tools and links for facultative use: www.palliatievezorgcopd.nl
Training session 1 (3 h)	Introductory information on the project and research
	Instruction on the Propal-COPD tool to identify the palliative phase in patients with COPD
	Multidimensional assessment (physical, psychological, social, spiritual)
	Communication training on advance care planning in COPD including roleplay with actors
	Non-pharmacological and pharmacological dyspnoea management based on the Breathing-Thinking-Functioning model^14^
Training session 2 (3 h)	Discussion current palliative care as organized in region vs desired palliative care
	Introductory information on implementing care pathway
	Filling in formats (who does what how and when) leading to first draft of regional action plan
	Assigning local implementation leaders
Completion of regional action plan	Agreement on who does what, how and when
Monitoring	Monitoring meetings on site
	Evaluation meetings with local implementation groups
*Integrated palliative care intervention*	
(1) Palliative care conversations	Consultation at outpatient clinic with patient and informal caregiver by pulmonologist and/or COPD nurse, including
	• Multidimensional assessment
	• Symptom management
	• Advance care planning
If needed	Follow up palliative care conversation(s)
	Specialist palliative care team consultation(s)
(2) Coordination and continuity	Individual care plan and documentation of treatment preferences
	Information exchange and collaboration with general practitioners and other involved professionals
	Regular multidisciplinary meetings
If a patient had died	
(3) Aftercare	Consultation with informal caregiver to evaluate care in the last phase
	Evaluation of the provided palliative care with all involved professionals

### Participants

Between May 2019 and August 2020, patients admitted to the hospital for an acute exacerbation were invited by a pulmonologist or nurse to participate and subsequently screened with the ProPal-COPD tool (see Box 1).^15^ Patients with a positive score were considered having palliative care needs and were included in the study. Initially, the previously published cut-off value of −1.362 was used.^15^ However, as the rate of patients with a positive score was lower than anticipated, it was deemed necessary to lower the cut-off value by one point to −2.4 after 6 months. Exclusion criteria for participation were the inability to complete questionnaires in Dutch, severe cognitive decline and being on the waiting list for lung transplantation (Table 2).

**Box 1.** ProPal-COPD tool.

The ProPal COPD tool was developed by Duenk et al.^15^ and consists of seven indicators: Medical Research Council (MRC) dyspnoea score of 5, Clinical COPD Questionnaire (CCQ) score >3, forced expiratory volume in 1 s lower than 30% predicted, presence of specific comorbidities, body mass index lower than 21 kg/m^2^ or weight loss (>10% in the last 6 months or >5% in last month), previous hospitalization for acute exacerbation in the last 2 years (last 2 years ⩾2 admissions or last year ⩾1 admission) and a negative answer to the surprise question (‘Would I be surprised if this patient were to die in the next 12 months?’).

**Table 2.** Inclusion and exclusion criteria of study participants.

Inclusion criteria	Exclusion criteria
Patient diagnosed with COPD	Inability to complete questionnaires in Dutch
Being admitted with an acute exacerbation COPD	Severe cognitive decline (e.g. dementia)
ProPal-COPD score positive (i.e. above cut-off value)	Being on the waiting list for lung transplantation

### Blinding

Complete blinding of participants for group allocation was impossible, but patients were not explicitly told whether their hospital was assigned to the intervention or control group. Further, healthcare providers of control regions were blinded for the ProPal-COPD score (whether positive and thus needing palliative care, or negative).

### Data collection

Demographics and patient-reported outcome measures were collected using a questionnaire at three time points. At baseline, patients completed a paper questionnaire during hospitalization. After 3 and 6 months, a follow-up questionnaire was sent to the patient’s home or email, depending on the patient’s preference. Patients were called by phone to remind them to complete the follow-up questionnaires. However, this was not always possible due to staff shortages in the research team. Medical record assessment was performed after 12 months to retrieve data on healthcare use, documentation of treatment preferences and date and place of death. Also, we assessed how many patients had received intervention components. Intervention patients who had had at least one palliative care conversation at the outpatient clinic with their pulmonologist and/or COPD nurse within 6 months after inclusion were considered to have received the intervention with fidelity.

### Outcome measures

The primary outcome was quality of life measured with the validated 46-item Functional Assessment of Chronic Illness Therapy-Palliative care (FACIT-Pal) scale.^16^ Total score ranges between 0 and 184, with a higher score indicating a better quality of life. Two subscores were calculated: the FACT-G sub score (a combination of the four general subscales on physical, social/family, emotional and functional well-being, consisting of 27 items) and the PALS sub score (the specific palliative care subscale, consisting of 19 items). Secondary outcomes were health-related quality of life (CCQ), spiritual well-being (FACIT–Spiritual Well Being scale (FACIT-Sp-12)), anxiety and depression symptoms (Hospital Anxiety and Depression Scale (HADS)), satisfaction with care received from the hospital and general practice, respectively (numerical rating scale (NRS) ranging from 0 to 10). Furthermore, the number of emergency department visits, hospital admissions (number and number of days) and intensive care unit (ICU) admissions were assessed. Also, we verified if any life-sustaining treatment preferences (e.g. cardiopulmonary resuscitation) had been documented. Lastly, the date and place of death of deceased patients were collected and whether any emergency department or hospital admission had occurred in the last month of life. We also intended to collect and analyse informal caregiver burden data. However, due to low recruitment rates and high non-response rates, the data obtained were insufficient to conduct analyses.

### Data analyses

Data cleaning and descriptive statistics were performed using SPSS, version 25, and outcome analyses were conducted using R software, version 3.6.2. We calculated that 347 participants were required to find an effect of minimum nine points at the primary outcome with an assumed standard deviation of 25, taking clustering at hospital level and a loss to follow-up of 10% into account.^13^ Primary and secondary outcomes were analysed using generalized linear mixed modelling with a normal distribution with identity link for continuous variables, negative binomial distribution with log link for count outcomes and log regression analysis for binary outcomes. A Hurdle model consisting of two parts (a binomial distribution with logit link and negative binomial distribution with log link) was used to compare the number of hospitalization days. The binomial part estimates the difference in the likelihood of having any hospitalization days by means of an odds ratio, while the negative binomial part estimates the ratio between the hospitalization days per time if larger than 0 using an incidence rate ratio. In the case of skewed residuals of continuous outcomes, bootstrapping was used. In all models, the baseline value of the outcome was entered as covariate and follow-up values as a dependent variable. To adjust for clustering, hospital region was entered as a random factor. The intraclass cluster coefficient was about zero for all outcomes, except for satisfaction with care from the hospital (0.031) and general practice (0.037). We checked for any unbalances in baseline characteristics and considered adjustment for these variables not required. Survival within 12 months between the two groups was analysed using a Kaplan-Meier plot and a Log Rank test. Differences between the two groups regarding the place of death and acute healthcare use in the last month of life were analysed using Chi-square tests. All outcomes were analysed using the intention-to-treat principle. Additionally, the occurrence of palliative care conversations in the intervention and control group was compared using a Chi-square test. A sensitivity analysis was done by limiting intervention participants to those who received one or more palliative care conversations at the outpatient clinic within 6 months after inclusion. All tests were two-sided, and *p*-values ⩽0.05 were considered statistically significant.

### Ethics approval and consent

All participants received oral and written study information and gave written informed consent. Ethical approval was granted by the Medical Ethics Committee of Arnhem-Nijmegen (file number 2018-4833) on 15 October 2018.

## Results

### Participant characteristics

**Figure 1.**
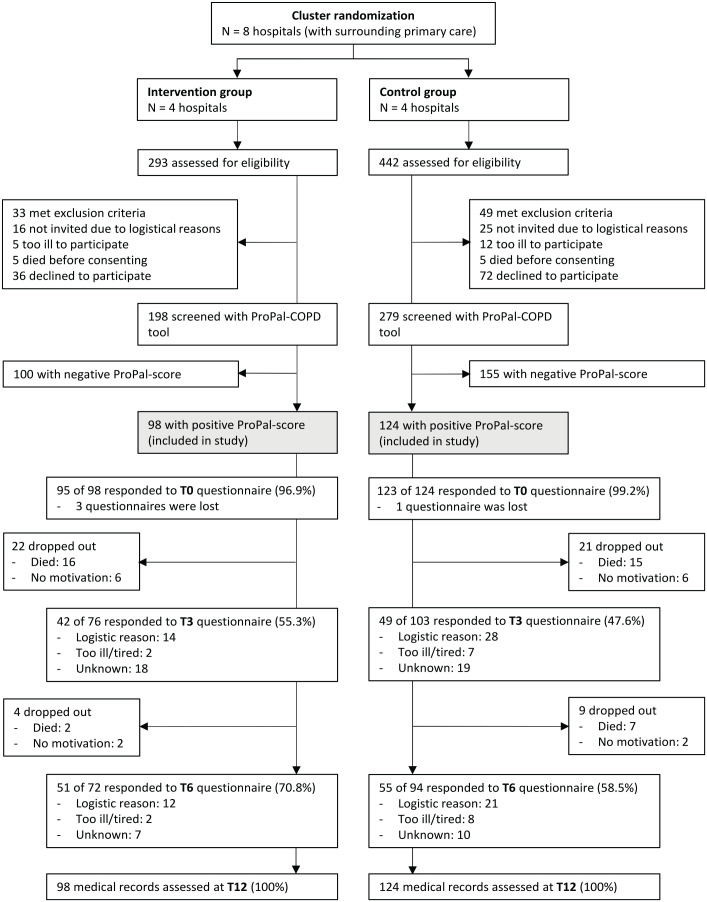
Flow diagram of inclusion of participants and response rates of questionnaires at baseline (T0), after 3 months (T3) and 6 months (T6).

Between May 2019 and August 2020, 735 patients admitted to the hospital for an acute exacerbation COPD were screened for eligibility (Figure 1). Of 477 consenting patients, 222 had a positive ProPal-COPD score and were included in the study, 98 in the intervention group and 124 in the control group. Fifty-six patients dropped out within 6 months after inclusion because of death (*n* = 40) or reluctance to complete the questionnaires (*n* = 16). At 3 and 6 months, 91 of 179 (50.8%) and 106 of 166 (63.9%) patients responded to the follow-up questionnaires. Dropout and non-response rates were similar across the two groups, and baseline characteristics of responders did not differ from non-responders. Table 3 shows the baseline characteristics of all participants and of those with at least one complete FACIT-Pal score during follow-up. On average, patients of the intervention group had a lower lung function, higher education level and, more often, one or more comorbidities; other characteristics did not differ significantly.

**Table 3.** Demographic- and clinical characteristics of participants in the intervention and control group and participants with at least one complete follow-up FACIT-Pal score.

	Intervention	Control	Intervention – complete scores	Control – complete scores
	*n* = 98	*n* = 124	*n* = 56	*n* = 61
*Demographic characteristics*				
Age in years, mean ± SD	69.4 ± 8.7	69.8 ± 9.1	67.2 ± 9.0	69.5 ± 8.6
Sex, female	57 (58.2)	75 (60.5)	35 (62.5)	36 (59.0)
Marital status				
Married	53 (57.6)	54 (44.3)	34 (64.2)	28 (46.7)
Unmarried	7 (7.6)	21 (17.2)	5 (9.4)	11 (18.3)
Divorced	11 (12.0)	21 (17.2)	4 (7.5)	9 (15.0)
Widow	21 (22.8)	26 (21.3)	10 (18.9)	12 (20.0)
Living situation				
Living alone	36 (39.1)	47 (47.5)	17 (32.1)	26 (44.1)
Living together	56 (60.9)	63 (52.5)	36 (67.9)	33 (55.9)
Place of living				
Home, without homecare	64 (70.3)	78 (64.5)	41 (78.8)	40 (66.7)
Home, with homecare	26 (28.6)	37 (30.6)	11 (21.2)	17 (28.3)
Residential home	1 (1.1)	4 (3.3)	0 (0.0)	3 (5.0)
Nursing home	0 (0.0)	2 (1.7)	0 (0.0)	0 (0.0)
Country of birth				
Netherlands	88 (95.7)	116 (95.9)	50 (94.3)	57 (95.0)
Other	4 (4.3)	5 (4.1)	3 (5.7)	3 (5.0)
Highest level of education				
No education or elementary school	15 (16.3)	30 (25.0)	6 (11.3)	11 (18.6)
Secondary school	19 (20.7)	40 (33.3)	12 (22.6)	21 (35.6)
Vocational education	48 (52.2)	40 (33.3)	29 (54.7)	20 (33.9)
Higher/university	10 (10.9)	10 (8.3)	6 (11.3)	7 (11.9)
*Clinical characteristics*				
Current smoker	19 (20.2)	31 (25.6)	10 (17.9)	12 (20.0)
Pack years, mean ± SD	40.7 ± 27.6	42.8 ± 27.4	35.8 ± 22.2	43.4 ± 30.8
FEV1 % of predicted, mean ± SD	36.6 ± 13.4	38.1 ± 15.5	34.8 ± 13.6	39.8 ± 15.4
GOLD stage				
1	(0.0)	1 (0.8)	0 (0.0)	0 (0.0)
2	17 (17.3)	22 (17.7)	9 (16.1)	13 (21.3)
3	31 (31.6)	45 (36.3)	18 (32.1)	24 (39.3)
4	48 (49.0)	52 (41.9)	29 (51.8)	23 (37.7)
Unknown	2 (2.0)	4 (3.2)		1 (1.6)
*ProPal-COPD tool indicators*				
MRC dyspnoea score = 5	71 (72.4)	103 (83.1)	42 (75.0)	53 (86.9)
CCQ score >3	72 (73.5)	92 (74.2)	43 (76.8)	50 (82.0)
Comorbidity	40 (40.8)	31 (25.0)	23 (41.1)	14 (23.0)
Non-curable malignancy	5 (5.1)	6 (4.8)	3 (5.4)	2 (3.3)
Cor pulmonale	14 (14.3)	8 (6.5)	10 (17.9)	3 (4.9)
Chronic heart failure	16 (16.3)	14 (11.3)	7 (12.5)	8 (13.1)
Diabetes with neuropathy	6 (6.1)	3 (2.4)	2 (3.6)	2 (3.3)
Renal failure	5 (5.1)	5 (4.0)	2 (3.6)	2 (3.3)
Previous hospitalization	50 (51.0)	70 (56.5)	29 (51.8)	37 (60.7)
BMI < 21 or weight loss	35 (35.7)	48 (38.7)	22 (39.3)	20 (32.8)
FEV1% of predicted <30%	33 (33.7)	40 (32.3)	23 (41.1)	17 (27.9)
Surprise question, negative	56 (57.1)	69 (55.6)	32 (57.1)	34 (55.7)

Data presented as percentage unless stated otherwise.

BMI: body mass index; FEV1: forced expiratory volume in the first second; MRC: Medical Research Council; SD: standard deviation.

### Intervention delivery

In the intervention group, an outpatient palliative care conversation occurred in 36 of 98 patients within 6 months after inclusion (36.7%). In eight patients, a conversation took place later than after 6 months. Reasons for no outpatient palliative care conversation were: transferral to a different care setting (primary care, rehabilitation centre or nursing home) (*n* = 9), postponement due to the COVID-19 pandemic (*n* = 6), death of patient before consultation took place (*n* = 9), reluctance of patient (*n* = 7) or psychiatric illness (*n* = 1), initially negative ProPal-score (*n* = 8) and unknown (*n* = 14).

In the control group, an outpatient palliative care conversation occurred in 4 of 124 patients within 6 months after inclusion (3.2%). The occurrence of these conversations was in the intervention group statistically significantly higher than in the control group with an odds ratio of 17.42 (95% CI: 5.93–51.17), *p* < 0.001.

### Outcomes

The FACIT-Pal score, the primary outcome, showed no difference between the intervention and control group in the intention-to-treat analysis (adjusted difference of 1.090 (95% CI: −5.440 to 7.600), *p* = 0.744). Also, no differences in secondary patient-reported outcome measures were found (Table 4). In the intervention group, the number of ICU admissions for COPD was lower (adjusted odds ratio of 0.212 (95% CI: 0.032–0.813), *p* = 0.047), and there was an indication of fewer hospitalizations for COPD (adjusted incidence rate ratio of 0.690 (95% CI: 0.462–1.026); *p* = 0.068). Other healthcare use outcome measures did not differ between the groups (Table 5).

**Table 4.** Response numbers and outcomes at baseline, after 3 and 6 months and differences between intervention and control group.

	*n*	Intervention group	*n*	Control group	Adjusted difference* (95% CI)	*p* Value
		Mean (SD)		Mean (SD)		
*Primary outcome*						
FACIT-Pal total					1.090 (−5.440 to 7.600)	0.744
Baseline	94	104.0 (19.3)	120	106.6 (23.7)		
3 months	38	108.4 (25.2)	43	111.0 (22.2)		
6 months	49	113.3 (22.6)	51	111.7 (22.8)		
*Secondary PROM outcomes*						
FACT-G subscore					2.010 (−2.180 to 6.150)	0.379
Baseline	93	58.7 (11.9)	120	60.2 (15.9)		
3 months	39	61.9 (14.3)	44	62.7 (14.4)		
6 months	48	65.8 (14.8)	51	64.1 (15.0)		
PALS subscore					−0.815 (−3.540 to 1.910)	0.562
Baseline	95	45.3 (8.8)	123	46.4 (9.5)		
3 months	40	46.3 (11.5)	44	48.2 (8.8)		
6 months	50	47.1 (9.3)	54	47.4 (8.7)		
CCQ day score**					−0.225 (−0.572 to 0.123)	0.211
Baseline	97	3.60 (0.9)	123	3.68 (1.1)		
3 months	41	3.03 (1.1)	48	3.38 (1.0)		
6 months	50	2.94 (1.0)	55	3.29 (1.0)		
HADS anxiety**					−0.591 (−1.810 to 0.629)	0.347
Baseline	95	8.9 (4.6)	120	8.5 (5.3)		
3 months	41	7.8 (4.5)	43	7.7 (5.0)		
6 months	49	6.8 (4.7)	54	6.6 (4.5)		
HADS depression**					−0.378 (−1.660 to 0.903)	0.566
Baseline	95	8.7 (4.1)	120	8.1 (4.4)		
3 months	41	8.3 (4.3)	43	8.3 (4.4)		
6 months	49	7.2 (4.3)	54	7.2 (4.5)		
FACIT-Sp-12					0.068 (−1.72 to 1.86)	0.941
Baseline	89	22.9 (7.2)	113	26.2 (9.4)		
3 months	38	22.4 (7.8)	44	25.4 (8.3)		
6 months	44	22.7 (6.6)	51	24.7 (6.9)		
Satisfaction with hospital care					0.254 (−0.593 to 1.130)	0.592
Baseline	91	7.9 (1.5)	118	8.0 (1.6)		
6 months	46	8.1 (1.3)	48	7.9 (2.1)		
Satisfaction with GP care					−0.215 (−1.130 to 0.685)	0.711
Baseline	87	7.2 (2.0)	118	7.3 (2.3)		
6 months	42	6.9 (2.5)	48	7.4 (2.4)		

CCQ: clinical COPD questionnaire; CI: confidence interval; FACIT-Pal: Functional Assessment of Chronic Illness Therapy Palliative care; FACT-G: Functional Assessment of Cancer Therapy General subscale; GP: general practitioner; HADS: Hospital Anxiety and Depression Scale; PALS: Palliative care subscale of the FACIT-Pal; PROM: patient-reported outcome measure.

* Adjusted for baseline levels and clustering. **Higher score indicates worse.

**Table 5.** Numbers of acute healthcare use 1 year before and 1 year after inclusion and differences between intervention and control group.

	Intervention group	Control group	Adjusted incidence rate ratio (95% CI)	*p* Value
	*n* = 98	*n* = 124		
	Mean (SD)	Mean (SD)		
Number of ED visits total			1.558 (0.444–5.471)	0.489
Before	0.38 (0.73)	0.31 (0.78)		
After	0.27 (0.57)	0.20 (0.57)		
Number of ED visits COPD			1.577 (0.394–6.307)	0.520
Before	0.32 (0.67)	0.20 (0.60)		
After	0.16 (0.47)	0.10 (0.38)		
Number of hospitalizations total			0.757 (0.472–1.213)	0.247
Before	0.95 (1.26)	1.23 (1.60)		
After	0.96 (1.38)	1.37 (1.74)		
Number of hospitalizations COPD			0.690 (0.462–1.026)	0.068
Before	0.65 (1.02)	0.77 (1.11)		
After	0.65 (1.03)	0.98 (1.41)		
Number of hospital days COPD*			0.585 (0.315–1.02)** 0.98 (0.717–1.29)	0.074 0.893
Before	4.85 (8.84)	5.50 (8.75)		
After	5.06 (8.48)	7.10 (10.07)		
			Adjusted odds ratio (95% CI)	*p* Value
Number of ICU admission total			0.520 (0.178–1.425)	0.216
Before	0.10 (0.30)	0.11 (0.37)		
After	0.10 (0.44)	0.21 (0.93)		
Number of ICU admission COPD			0.212 (0.032–0.813)	0.047
Before	0.08 (0.28)	0.14 (0.55)		
After	0.02 (0.14)	0.09 (0.29)		
Patients with life-sustaining treatment preferences documented, *n* (%)	54 (55.1%)	61 (49.2%)	1.227 (0.720–2.092)	0.452

CI: confidence interval; COPD: chronic obstructive pulmonary disease; ED: emergency department; GP: general practitioner; ICU: intensive care unit.

* For the number of hospital days COPD, the analysis was done using a Hurdle model, which gives two outcomes: the odds ratio for having any hospitalization days and an incidence rate ratio for the ratio of hospitalization days per time (if >0). **Adjusted odds ratio.

**Figure 2.**
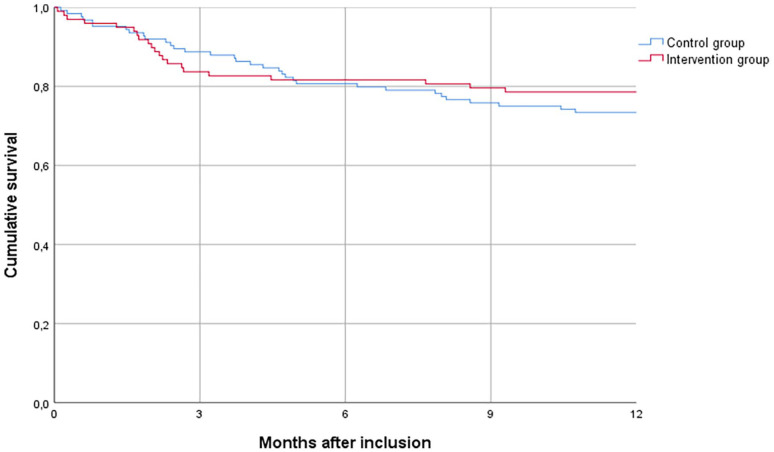
Kaplan-Meier plot of survival of the intervention and control group. Log Rank test: *p* = 0.458.

One year after inclusion, 54 patients (24.3%) had died; 21 in the intervention group and 33 in the control group. The Kaplan-Meier curve is shown in Figure 2. Survival did not differ between intervention and control patients (*p* = 0.458). Place of death and acute healthcare use in the last month of life did not differ between the two groups (Table 6).

**Table 6.** Place of death and acute healthcare use in last month of life of participants in intervention and control group.

	Intervention group	Control group	Odds ratio (95% CI)	*p* Value*
Patients who died	21/98 (21.4%)	33/124 (26.6%)	0.75 (0.40–1.41)	0.372
In-hospital death	5/21 (23.8%)	12/33 (36.4%)	0.55 (0.16–1.87)	0.336
Emergency department or hospital admission in last month	14/21 (66.6%)	22/33 (66.6%)	1.00 (0.31–3.19)	1.000

* *p*-Values based on Chi-square test.

In the sensitivity analysis, limiting the intervention group to patients that received at least one outpatient palliative care conversation within 6 months (*n* = 36), findings regarding the primary outcome and other secondary outcomes were similar, except for ICU admissions and documentation of life-sustaining treatment preferences (Supplemental Tables 1 and 2). The effect on the number of ICU admissions disappeared (adjusted odds ratio of 0.591 (95% CI: 0.088–2.352), *p* = 0.508). Life-sustaining treatment preferences were more often documented in intervention patients than in controls (adjusted odds ratio of 4.817 (95% CI: 1.930–12.026), *p* = 0.001).

## Discussion

### Main findings

In this cluster randomized controlled trial, we assessed the effectiveness of palliative care components integrated into regular COPD care. We found no effects on quality of life nor other patient-reported outcome measures. However, intervention patients were less frequently admitted to the ICU than control patients, and there was a strong indication for fewer hospital admissions. Sensitivity analyses did not corroborate these findings but showed that the intervention increased documentation of life-sustaining treatment preferences.

### Interpretation of findings

Similar to our study, a recent systematic review found no effect of palliative care interventions on the quality of life of patients with COPD; effects on acute healthcare use were inconclusive.^9^ It contrasts, however, with palliative care intervention studies in patients with cancer or chronic heart failure, in whom improved quality of life and less acute healthcare use was demonstrated.^6,17,18^

Our findings could be explained in several ways. First, we did not reach sufficient statistical power to detect effects on the primary outcome measure reliably. To increase recruitment, we lowered the cut-off value of the ProPal-COPD tool after 6 months, but then the COVID-19 pandemic again hampered recruitment rates.

Second, implementation was suboptimal. Because of several reasons, such as the COVID-19 pandemic, a significant part of the intervention group did not receive an outpatient palliative care conversation. Also, coordination and continuity of care between hospital and primary care remained challenging. The barriers and facilitators to successful implementation we encountered have been published in our process evaluation article separately.^19^ Nevertheless, our rate of 37% is comparable to the average rate (33%) found across advance care planning intervention studies.^20^

Effects at the provider’s level tended to be more prominent in our study, probably because our implementation strategy was at healthcare provider level: they were trained and guided to implement palliative care components. Indeed, many more outpatient palliative care conversations took place than in the control group, and treatment preferences were documented more often. Also, we found that self-efficacy in palliative care provision increased in trained healthcare providers.^19^ Thus, although no effects were found at patient level, our implementation strategy effectively changed providers’ behaviour.

Third, quality of life and other well-being outcomes are broad constructs influenced by many factors. The potential to improve overall quality of life may be limited in advanced organ failure, and the fluctuations in the disease course further complicate such outcome measurements.^21^ It is probable that our intervention, mainly consisting of a single palliative care conversation, was insufficiently intensive to improve clinical outcomes. Also, these conversations may affect only certain aspects of quality of life. In previous palliative care trials, positive effects were found on outcomes related to ‘coping with COPD’: self-management,^22^ mastery of breathlessness^23^ and the impact subscale of the St. George’s Respiratory Questionnaire (SGRQ).^24^ In interviews we held to assess the implementation process, healthcare providers indicated to highly value the intervention because of the positive effects of the palliative care conversations for their patients. According to them, patients expressed that knowing what would happen if the disease worsened and the care possibilities provided them clarity and peace of mind.^19^ Feeling better equipped to cope with a severe chronic illness affects the patient’s quality of life but may not be reflected in an overall quality of life measure.

Although we did not find an effect on quality of life, our study in COPD is the first controlled study that found a lower rate of ICU admissions in the palliative care group,^9^ and is the second controlled trial that found a non-significant trend for fewer hospital admissions.^25^ Even though these findings were not corroborated in the sensitivity analysis, trained healthcare providers of the intervention group may have become more aware of the disadvantages of invasive treatments making them more reluctant to refer patients to the ICU. The COVID-19 pandemic may have reinforced this reluctance. As intervention patients had more often comorbidities, this could also have caused a lower rate of ICU admissions found in this group. As ICU admissions contribute most to COPD-related healthcare costs,^26^ palliative care may lower healthcare costs considerably, making it attractive to policymakers and healthcare insurers to encourage and reimburse palliative care.

### Strengths and limitations

This study is the first large randomized controlled trial assessing the effectiveness of palliative care integrated into regular COPD care. As part of a hybrid type 2 effectiveness-implementation study,^27^ the implementation was done in a real-world setting without additional human and financial resources and thus reflected naturalistic findings. Also, the multicentre design makes our findings generalizable to other hospital regions. Furthermore, we chose for cluster-level randomization to prevent contamination between the intervention and control group.

However, our study also has limitations. Next to insufficient study power, we had a high rate of missing data due to the death of participants and high non-response to follow-up questionnaires. Missing data are expected in palliative care studies and increase with more items, quality of life questionnaires and longer follow-up time.^28^ Consistent with previous studies in this patient population,^24,29^ completing the questionnaire proved to be burdensome to some patients, and specific questions of the FACIT-Pal questionnaire were perceived as confrontational. Frequently, patients needed help from a healthcare provider to complete the questionnaire, as reflected by the high completion rate of baseline questionnaires during hospitalization and low completion rates of follow-up questionnaires that had to be filled out at home. If sufficient resources are available, future studies could involve a research nurse administering the questionnaire at the patient’s home to minimize missing data.^30^ However, since both groups’ attrition rates were similar and responders’ characteristics did not significantly differ from those of non-responders, the risk of poor internal validity is low.

## Conclusions

The effect of integrated palliative care on clinical outcomes in patients with COPD remains inconclusive. We found no evidence that palliative care improves quality of life in patients with COPD, but it can potentially reduce ICU admissions. Better implementation of palliative care components is needed to enhance reliable effect evaluation. Future research should consider using an outcome measure related to coping with COPD that is easy to complete by patients with advanced disease.

## Supplemental Material

sj-pdf-1-pmj-10.1177_02692163231165106 – Supplemental material for The effect of an integrated palliative care intervention on quality of life and acute healthcare use in patients with COPD: Results of the COMPASSION cluster randomized controlled trial Click here for additional data file. Supplemental material, sj-pdf-1-pmj-10.1177_02692163231165106 for The effect of an integrated palliative care intervention on quality of life and acute healthcare use in patients with COPD: Results of the COMPASSION cluster randomized controlled trial by Johanna Broese, Rianne MJJ van der Kleij, Els ML Verschuur, Huib AM Kerstjens, Ewald M Bronkhorst, Yvonne Engels and Niels H Chavannes in Palliative Medicine
