# Supplementary material for: The effect of an integrated palliative care intervention on quality of life and acute healthcare use in patients with COPD: Results of the COMPASSION cluster randomized controlled trial
Source: Palliat Med. 2023 Mar 31;37(6):844–55. doi: 10.1177/02692163231165106 (PMC10227092; doi:10.1177/02692163231165106)
Supplement: sj-pdf-1-pmj-10.1177_02692163231165106 – Supplemental material for The effect of an integrated palliative care intervention on quality of life and acute healthcare use in patients with COPD: Results of the COMPASSION cluster randomized controlled trial [file sj-pdf-1-pmj-10.1177_02692163231165106.pdf]

Supplementary Table 1. Outcomes of the sensitivity analyses limiting intervention patients to those who received the intervention within 6 months (n=36).

	n	**Intervention patients who received intervention** Mean (SD)	**Adjusted difference (95%-CI)**	**P value**
**Primary outcome**				
FACIT-Pal total			-0.782 (-9.380 – 7.830)	0.860
Baseline	35	106.7 (17.4)		
3 months	16	105.2 (32.0)		
6 months	20	119.0 (19.8)		
**Secondary PROM outcomes**				
FACT-G subscore			1.590 (-3.930 – 6.980)	0.568
Baseline	35	60.8 (10.3)		
3 months	16	61.2 (18.1)		
6 months	20	70.7 (12.3)		
PALS subscore			-1.860 (-5.410 – 1.690)	0.311
Baseline	35	45.9 (8.4)		
3 months	16	44.0 (14.5)		
6 months	20	48.4 (8.0)		
CCQ day score			-0.115 (-0.569 – 0.338)	0.622
Baseline	36	3.6 (0.9)		
3 months	16	3.2 (1.2)		
6 months	20	2.8 (1.0)		
HADS anxiety			-0.843 (-2.450 – 0.763)	0.309
Baseline	35	7.9 (4.4)		
3 months	16	8.0 (4.2)		
6 months	20	4.9 (4.1)		
HADS depression			-0.337 (-2.050 – 1.380)	0.703
Baseline	35	7.7 (3.8)		
3 months	16	8.8 (4.4)		
6 months	20	6.1 (4.2)		
FACIT-Sp-12			-1.100 (-3.550 – 1.340)	0.384
Baseline	31	22.4 (6.2)		

3 months	15	21.2 (8.6)		
6 months	17	23.4 (5.4)		
Satisfaction with hospital care			0.360 (-0.884 – 1.610)	0.595
baseline	34	8.0 (1.3)		
6 months	18	8.2 (1.2)		
Satisfaction with GP care			0.135 (-1.040 – 1.340)	0.847
Baseline	31	7.6 (1.3)		
6 months	16	7.6 (2.2)		
**Acute healthcare use**			**Adjusted Incidence Rate Ratio (95%-CI)**	**P value**
Number of ED visits total			0.767 (0.166 – 3.552)	0.735
Before	36	0.36 (0.83)		
After	36	0.31 (0.71)		
Number of ED visits COPD			1.104 (0.259 – 4.712)	0.893
Before	36	0.14 (0.35)		
After	36	0.11 (0.32)		
Number of hospitalizations total			0.755 (0.472 – 1.208)	0.241
Before	36	1.19 (1.37)		
After	36	1.03 (1.34)		
Number of hospitalizations COPD			0.780 (0.455 – 1.320)	0.361
Before	36	0.83 (1.11)		
After	36	0.81 (1.14)		
Number of hospital days COPD*			0.780 (0.356 – 1.570)**<br>0.994 (0.664 – 1.430)	0.512<br>0.975
Before	36	5.58 (8.13)		
After	36	6.50 (9.69)		
			**Adjusted Odds Ratio (95%-CI)**	**P value**
Number of ICU admission total			0.580 (0.112 – 2.306)	0.469
Before	36	0.06 (0.23)		
After	36	0.08 (0.28)		
Number of ICU admission COPD			0.591 (0.088 – 2.352)	0.508
Before	36	0.03 (0.17)		

After	36	0.06 (0.23)		
Patients with life-sustaining treatment preferences documented, n(%)	36	28 (77.8%)	4.817 (1.930 – 12.026)	0.001

Abbreviations: CCQ, clinical COPD questionnaire; CI, confidence interval; COPD, chronic obstructive pulmonary disease; ED, emergency department; FACIT-Pal, Functional Assessment of Chronic Illness Therapy Palliative care; FACT-G, Functional Assessment of Cancer Therapy General subscale; GP, general practitioner; HADS, Hospital Anxiety and Depression Scale; ICU, intensive care unit; PALS, Palliative care subscale of the FACIT-Pal; PROM, patient reported outcome measure.

*For the number of hospital days COPD, the analysis was done using a Hurdle model, which gives two outcomes: the odds ratio for having any hospitalization days and an incidence rate ratio for the ratio of hospitalization days per time (if >0). **Adjusted odds ratio.

Supplementary Table 2. Sensitivity analyses of place of death and acute healthcare use in last month.

	Intervention patients who received intervention	Control group	Odds ratio (95%-CI)	P value*
Patients who died	4/36 (11.1%)	33/124 (26.6%)	0.345 (0.113 – 1.049)	0.052
In-hospital death	1/4 (25%)	12/33 (36.4%)	0.583 (0.054 – 6.251)	0.653
Emergency department or hospital admission in last month	2/4 (50%)	22/33 (66.6%)	0.500 (0.062 – 4.040)	0.510

* P-values based on Chi square test.
